# International Clinics—A Quarterly of Clinical Lectures on Medicine, Etc.

**Published:** 1895-02

**Authors:** 


INTERNATIONAL CLINICS: A QUARTERLY OF CLINICAL LECTURES ON MEDICINE, NEUROLOGY, SURGERY, GENITO-URINARY SURGERY, GYNÆCOLOGY OBSTETRICS, OPHTHALMOLOGY, LARYNGOLOGY, PHARYNGOLOGY, RHINOLOGY, OTOLOGY, AND DERMATOLOGY, BY PROFESSORS AND LECTURERS IN THE LEADING MEDICAL COLLEGES OF THE UNITED STATES, GERMANY, FRANCE, GREAT BRITAIN, AND CANADA. Edited by Judson Daland, M. D., (University of Pennsylvania) Philadelphia, Instructor in Clinical Medicine and Lecturer on Physical Diagnosis in the University of Pennsylvania, Assistant Physician to the Hospital of the University of Pennsylvania, Physician to the Philadelphia Hospital, Fellow of the College of Physicians of Philadelphia; J. Mitchell Bruce, M. D., F. R. C. P., London, England, Physician to and Lecturer on the Principles and Practice of Medicine in the Charing Cross Hospital; David W. Finlay, M. D., F. R. C. P., Aberdeen, Scotland, Professor of Practice of Medicine in the University of Aberdeen, Physician to and Lecturer on Clinical Medicine in the Aberdeen Royal Infirmary, Consulting Physician to the Royal Hospital for Diseases of the Chest, London. Volume III, Fourth Series 1894. Philadelphia: J. B. Lippincott Company. 1894.

The "Clinics" have been before the profession since 1890, and have proved of great use. It is a great pleasure as well as a privilege for the poor physicians to read the lectures of so many distinguished teachers, without incurring the expense of leaving home and of leaving the field of practice. It is a greater pleasure as well as a greater privilege for those who have heard some of these teachers to be able to continue under their instruction through the medium of the *International Clinics*. Cases of all kinds are reported and it is rare indeed that the practitioner fails to find the report of a case that matches one that is puzzling him in practice.
J. McF. G., Jr.

